# Cytokines in Endocrine Dysfunction of Plasma Cell Disorders

**DOI:** 10.1155/2017/7586174

**Published:** 2017-06-27

**Authors:** Eva Feigerlová, Shyue-Fang Battaglia-Hsu

**Affiliations:** ^1^CHU de Poitiers, Service d'Endocrinologie, Pole DUNE, Poitiers, France; ^2^Université de Poitiers, UFR Médecine Pharmacie, Poitiers, France; ^3^INSERM, CIC 1402 & U1082, Poitiers, France; ^4^INSERM U954, Nutrition Génétique et Exposition aux Risques Environnementaux, Medical Faculty, University of Lorraine and Regional University Hospital Center of Nancy, Vandœuvre-lès-Nancy, France

## Abstract

Monoclonal gammopathies (MG) are classically associated with lytic bone lesions, hypercalcemia, anemia, and renal insufficiency. However, in some cases, symptoms of endocrine dysfunction are more prominent than these classical signs and misdiagnosis can thus be possible. This concerns especially the situation where the presence of M-protein is limited and the serum protein electrophoresis (sPEP) appears normal. To understand the origin of the endocrine symptoms associated with MG, we overview here the current knowledge on the complexity of interactions between cytokines and the endocrine system in MG and discuss the perspectives for both the diagnosis and treatments for this class of diseases. We also illustrate the role of major cytokines and growth factors such as IL-6, IL-1*β*, TNF-*α*, and VEGF in the endocrine system, as these tumor-relevant signaling molecules not only help the clonal expansion and invasion of the tumor cells but also influence cellular metabolism through autocrine, paracrine, and endocrine mechanisms. We further discuss the broader impact of these tumor environment-derived molecules and proinflammatory state on systemic hormone signaling. The diagnostic challenges and clinical work-up are illustrated from the point of view of an endocrinologist.

## 1. Introduction

Plasma cell disorders are characterized by disproportionate proliferation of single clones of B cells that give rise to both structurally and electrophoretically homogeneous (monoclonal) immunoglobulins (either intact or subunits only) in body fluids such as urine and serum. Their classification is made based on both clinical symptoms and coexisting pathological conditions, including monoclonal gammopathy of undetermined significance (MGUS), malignant plasma cell disorders (such as multiple myeloma (MM)), progressive and symptomatic heavy-chain diseases, and nonhereditary primary systemic amyloidosis [1]. Depending on the type of plasma cell disorders, the treatment strategy varies. For example, while no treatment is required for MGUS except a regular follow-up, many treatment solutions are possible for MM, which include chemotherapy, stem cell transplants, and radiation therapy, as well as the administration of corticosteroids, proteasome inhibitors, and immunomodulatory drugs such as thalidomide and lenalidomide [2].

Diverse endocrinopathies occur in patients with plasma cell disorders [3–6]. In some patients, instead of the signs typically observed in MG (such as lytic bone lesions, hypercalcemia, anemia, and renal insufficiency), the most revealing symptoms are those of a dysfunctional endocrine system, thus confounding the diagnosis of MG—an especially likely situation when the presence of monoclonal protein (M-protein) is very weak and the serum protein electrophoresis (sPEP) profile appears normal. One such disease scenario is the rather rare POEMS syndrome, which is a paraneoplastic syndrome with key manifestations of polyneuropathy, organomegaly, endocrinopathy, monoclonal gammopathy, and skin changes [7]. A pathophysiological link between the endocrinopathy and the underlying plasma cell disorder (PCD) is not well understood. It is plausible that both the plasma cell-derived factors and the microenvironment of these cancer cells participate in causing MG-related endocrine dysfunction [3].

Here, we summarize the available pathophysiological mechanisms linking endocrinopathies to MG reported in the literature. To illustrate possible diagnostic difficulties faced by endocrinologists, we use one case of POEMS syndrome with severe acute adrenal insufficiency to demonstrate the clinical course and the diagnostic challenge.

## 2. Endocrine Dysfunction in Monoclonal Gammopathy

POEMS syndrome is a rather rare paraneoplastic disorder, with only a limited number of retrospective series reported so far [8, 9]. These studies revealed that the endocrine dysfunction in POEMS syndrome patients can be both central and peripheral [10, 11]. The most frequent presentations are related to hypogonadism, thyroid dysfunction, and impaired glucose metabolism. Adrenal insufficiency has also been described in both the American and Japanese patients [9, 11] (for details, see Table 1). In cases where clinical symptoms of endocrinal dysfunction are much more prominent than the signs of polyneuropathy, a delayed diagnosis may arise from a misled diagnostic work-up [12].

Although the mechanistic link between cancerous plasma cells and endocrine dysfunction remains to be elucidated, it is believed that these abnormalities are not the consequences of structural damages to the endocrinal tissues since not only structurally intact endocrine glands have been found in autopsy samples [13]; a functional recovery is achievable upon treatments [14]. The present evidence does not support the hypothesis of autoimmunity against endocrine tissues as no immunoglobulin binding was found in the nerve tissue of POEMS patients exhibiting polyneuropathy [10] and in the parathyroid adenoma tissue of the MG patients with primary hyperparathyroidism (PHPT) [3, 15]. In addition, as shown in Table 1, monoclonal gammopathy has been identified in several cases of chronic thyroiditis without signs of autoimmunity [4–6].

## 3. Clinical Course and Diagnostic Challenges: Lessons from a Descriptive Case Study

To illustrate, we use an example of a patient with predominant endocrine symptoms. A 31-year-old male was admitted to the hospital with an acute adrenal insufficiency. Physical examination uncovered a marked hyperpigmentation on sun-exposed areas and predominant sensory neuropathy of the upper and lower limbs. The past medical history showed a 6-month-long episode of tiredness, weight loss, and erectile dysfunction. Abdominal computed tomography scan evidenced normal adrenal glands. The hormonal findings are summarized in Table 2. There were no signs of autoimmunity. Serum protein electrophoresis evidenced an apparent increase in polyclonal gamma globulins. Long-chain fatty acids were normal ruling out adrenoleukodystrophy. The patient was discharged from the hospital care on hydrocortisone (20 mg/day) and fludrocortisone (50 *μ*g/day) supplementation.

Few months later, the patient started to suffer from weight loss, diarrhea, and fatigue despite an increased daily hydrocortisone dose at 30 mg/day. Physical examination showed a slim male (BMI = 18 kg/m^2^) with a heart rate of 90 bpm, hypotension (70/60 mmHg), and peripheral edema. The patient presented also symmetric facial lipoatrophy on the Bichat fat pad, hypertrichosis on the lower limbs, acrocyanosis, white nails, and digital clubbing. Cardiac ultrasound disclosed four-chamber dilatation, global hypokinesis, and reduced ejection fraction at 38%. Thyroid-stimulating hormone was slightly elevated (Table 2). Thyroid ultrasonography was normal. Low levels of vitamins (B6, B9, B12, C, and E) and coagulation factors (V, VII, and X) were evidenced.

**Figure 1:**
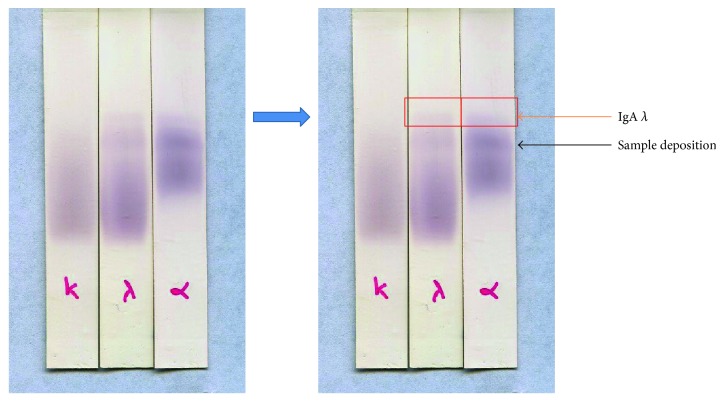
Immunoblot of the serum protein electrophoresis (sPEP) in the reported case. The red square indicates the identification of a low-level monoclonal protein IgA-*λ* using immunoblot of the sPEP. Its presence went unperceived in routine sPEP due to its very limited quantity masked by the dominant presence of beta-2-globulins. Fixation was performed at a 1/50 serum dilution. The labels below each track indicate the antibody used to reveal the Ig (from left to right): *κ*—light chain kappa, *λ*—light chain lambda, and *α*—heavy chain alpha (IgA).

Eighteen months later, the fundoscopic examination realized for frequent headaches demonstrated a bilateral papillary edema. At this point, a monoclonal protein of the type IgA-*λ* was uncovered by manually probing the immunoblots of sPEP (Figure 1()). Altogether, the clinical course and the laboratory test findings favored the diagnosis of POEMS syndrome (Table 3). Ensuing investigations unveiled a markedly increased serum VEGF (2820 pg/ml, ref < 500), normal interleukin-6 (IL-6) level (3.5 pg/ml, ref < 10), and normal bone marrow cytogenetics without osteosclerotic bone lesions. Surprisingly, the patient had no classical signs of MG-relevant organ or tissue impairment. The diagnosis of the POEMS syndrome was eventually established 18 months after the first clinical manifestation, and the treatment with a VEGF inhibitor lenalidomide along with dexamethasone was started. Two months later, the improvements in hormonal profile were observed Table 2.

## 4. How Do the Signaling Factors Work to Produce Endocrine Dysfunction in Plasma Cell Disorders?

Recent data indicate that endocrine dysfunction in patients with MG may be related to the production of growth factors as well as of cytokines such as IGFBP-2 and IGFBP-3, VEGF, IL-1*β*, IL-6, and TNF-*α* by tumor cells or their microenvironment [16–24]. Being transportable via the blood circulation, these molecules not only exert local proangiogenic and proproliferation effects on the tumor cells themselves and their immediate environments [25, 26] but also influence cellular metabolism at systemic level through autocrine, paracrine, and endocrine pathways [27, 28]. Potential mechanisms linking MG and endocrine dysfunction are summarized in Table 4, with examples of the factors secreted. The release of multiple mobile signaling factors warrants their complex interactions with multiple target cells/tissues such as osteoblasts and osteoclasts (for more information see [22, 29, 30]). The complexity of these interactions can arise from the fact that some of these molecules may enhance certain hormone signaling, while some others may inhibit the exact same interactions simultaneously. In addition, as such interactions are often tissue-dependent, the overall outcomes in patients can be unpredictable. Take, for example, the steroidogenesis; it can be suppressed by cytokines such as IL-1, IL-2, IL-6, and TNF-*α* in testicular and ovarian tissues [31, 32], but it can also be stimulated by the same cytokines in the adrenal gland. In the latter tissue, the multiple inputs from both the positive (tumor growth factor-*β*1) and the negative signaling molecules (interleukins) have been shown to reduce overall cortisol and aldosterone production in human adrenocortical cell line NCI-H295R [33]. Another example includes the role of IL-6 in pituitary; while it enhances the proliferation of tumor cells, it also inhibits the growth of normal cells [34, 35]. This is the reason why although it may seem logical to target the signaling network of cytokine and growth factor for alleviating endocrine dysfunction related to MG, no conclusive evidence has been obtained so far with interventions aiming to modulate the levels of cytokines and growth factors in POEMS syndrome patients [36]. For instance, VEGF has been related to extravascular overload [37] and neuronal damage in these patients [38]. However, no correlation has been evidenced either between VEGF and thyroid hormone level or between VEGF level and testicular function [14, 39].

## 5. Role of IL-6/Soluble IL-6 Receptor Signaling in Endocrine Dysfunction

**Figure 2:**
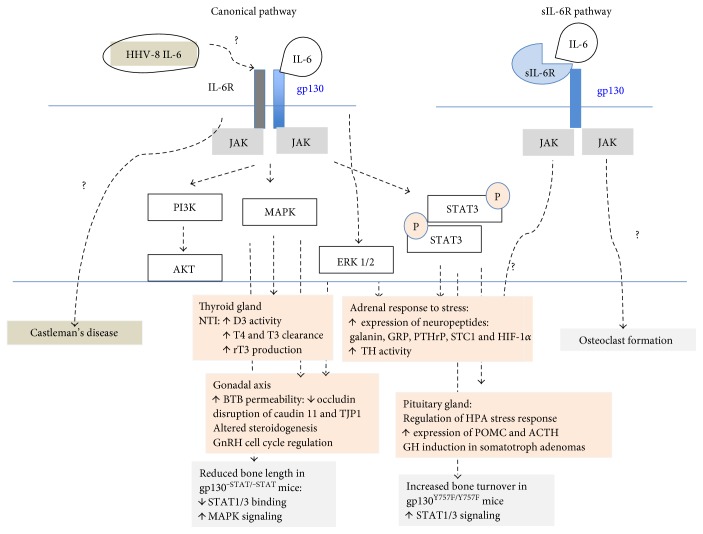
Overview of IL-6/soluble IL-6 receptor signaling at the nexus of endocrine function: illustrative examples. Binding of IL-6 to IL-6R and gp130 receptor complex leads to activation of Janus kinase- (JAK-) dependent pathways including mitogen-activated protein kinase (MAPK), protein kinase B (AKT)-phosphatidylinositol 3-kinase (PI3K), and signal transducer and activator of transcription 3 (STAT3). The IL-6 signaling mediated via a soluble IL-6 receptor (sIL-6R) leads to binding of IL-6/sIL-6R complex to gp130 triggering similar intracellular pathways (adapted from [30, 44, 45, 49]). In the states of NTI (nonthyroidal illness), an increase in rT3 production is a consequence of increased D3-mediated T3 (and T4) clearance through activation of the MAPK pathway [69]. Stress-induced HPA axis activation and GH-induced somatotroph adenoma growth are mediated via the STAT3 pathway [107]. In bovine chromaffin cells exposed to stress, IL-6 leads to activation of ERK 1/2 and STAT3 pathways and consequently to increased activity of tyrosine hydroxylase (TH), a rate-limiting enzyme in catecholamine synthesis, and to upregulation of downstream targets including secreted neuropeptides galanin, PTH-related peptide (PTHrP), G-protein-coupled receptor (GPR), stanniocalcin-1 (STC1), and hypoxia-inducible factor 1*α* (HIF-1*α*) [85]. The signaling via gp130 regulates the gonadal axis via activation of ERK and MAPK pathways on multiple levels: cycle regulation and proliferation of GnRH cells, impaired steroidogenesis, and increased permeability of blood-testis barrier (BTB) (through downregulation of occludin and delocalization of claudin 11 and tight junction protein 1 (TJP1)) [77, 115, 116]. Bone phenotype is illustrated through a rodent mutation model, where JAK/STAT and the SHP2/MAPK signaling regulate bone turnover and closure of the growth plate [56]. Soluble IL-6R seems to regulate osteoclast development in vitro; however, its relevance in vivo is unclear [92].

In MG, interleukin-6 (IL-6) produced by different cell types within the tumor microenvironments (including reactive stromal cells, tumor cells, and macrophages) is believed to stimulate the proliferation of plasma cells and play a critical role in the promotion of angiogenesis both directly and indirectly through enhanced VEGF secretion [40–43]. Mechanistic studies suggest that the underlying pathophysiological events are related to the activation of the pleotropic cell surface receptor gp130 [53, 57, 58] (Figure 2). Upon binding to the IL-6 dimeric receptor (composed of both gp130 and IL-6R subunits), IL-6 and its receptor form a hexameric ligand-bound complex (IL-6/IL-6R/gp130 in 2 : 2 : 2 stoichiometry) [44] capable of activating various intracellular signaling pathways including the canonical JAK/STAT (Janus kinase/signal transducers and activators of transcription) pathway, the PI3K/Akt pathway, and the SHP-2/JAK-dependent Ras/Raf-MAPK pathway [45–47]. Alternatively, in cells that do not express IL-6R subunit of the receptor complex, IL-6 can also exert its function via binding first to a soluble IL-6 receptor (sIL-6R); the transportable IL-6-sIL-6R complex, upon arriving to cells expressing only the pleiotropic cell surface receptor gp130, can then form a noncanonical gp130-containing ligand-receptor complex to trigger similar intracellular signaling pathways. As the expression in tissue of gp130 is ubiquitous [48], all cells are expected to respond to IL-6; this highlights thus potential far-reaching effects of IL-6 on cellular signaling and tissue function (for more details, see a recent review by Hunter and Jones [49]).

Incidentally, gp130 is a common signal transducer of the receptor complex for diverse cytokines commonly referred to as gp130 cytokines. This group of cytokines includes the homologs of IL-6 produced by virus such as human herpes virus-8 (HHV-8 IL-6) and rhesus macaque rhadinovirus (Rm IL-6) [50]. Interestingly, previous studies have suggested that these IL-6 homologs may exist in POEMS patients as the antibody to HHV-8 and the DNA of HHV-8 are both found in patients with POEMS syndrome [51–53] associated with Castleman's disease. Experimental evidence showed that HHV-8 IL-6 mimics the action of human IL-6 via binding directly to gp130 in the absence of IL-6R (the alpha receptor of IL-6) [54]. For the reported case present above, the HHV-8 serologic testing is negative and IL-6 level normal. However, we cannot rule out either the presence of other IL-6 family cytokines such as IL-11, oncostatin M, and leukemia inhibitory factor or the presence of soluble forms of IL-6 and gp130, which could activate IL-6 transsignaling.

IL-6 has been referred to as the most “endocrine” of all cytokines [55] as its level is regulated by hormones including glucocorticoids, estrogen, and catecholamine, and it exerts diverse effects on endocrine tissues. Table 4 summarizes the mechanistic links revealed so far between IL-6 pathways and endocrine dysfunction, excluding diabetic conditions. Up to date, the action of IL-6 in bone metabolism is best understood. IL-6 was shown first to have principally bone-resorptive effects but can enhance osteoformation in situations when bone turnover increases. Based on the study published by Sims et al. [56], the apparent shift in bone metabolism arises from two competing intracellular signaling pathways mediated by the signal transducer of the IL-6 receptor complex, gp130. Sims and coworkers used two different mutant mice to dissociate the two signaling pathways acting on chondrocyte, osteoclast, and osteoblast. One of the mutants gp130^ΔSTAT/ΔSTAT^ is a C-terminal-truncated mutant missing the STAT1/3 binding and activation domain; it retains, however, its capacity to activate the SHP2/Ras/MAPK signal cascade pathway; mechanistically, the shift from STAT1/3 to MAPK activity is achieved through the impaired activation of SOCS3 by STAT3, leading to altered docking of SOCS3 to the p-Y757-SHP-2 site of gp130 [56]. The other mutant gp130^Y757F/Y757F^ is with the point mutation Y757F; this particular mutation selectively blocks the activation of SHP2/Ras/MAPK but retains the capacity to signal through STAT1/3. The presentation of the distinct phenotypes of these mouse mutants led to the conclusion that JAK/STAT and SHP2/Ras/MAPK signaling pathways participate in differential regulation of bone growth and bone homeostasis. For example, the fact that gp130^STAT/ΔSTAT^ mice had no change in bone remodeling, bone turnover, and bone structure, but exhibited reduced chondrocyte proliferation and bone size, and also suffered from premature growth plate closure suggested that the gp130/STAT pathway plays a crucial role in maintaining a high level of growth plate chondrocyte proliferation during skeletal growth. On the contrary, the high-turnover phenotype of the mutant gp130^Y757F/Y757F^ mice was due to the presence of high number of both osteoblasts and osteoclasts and consequently had only a slight net bone loss [56]. To assess the role of IL-6 on gp130 signaling, these authors crossed gp130^Y757F/Y757F^ mice with IL-6^−/−^ mice to produce compound mutant mice. They observed that the double mutant had further reduction in bone mass despite absence of bone abnormality, suggesting thus that IL-6 regulates the gp130-dependent SHP2/Ras/MAPK effects on bone formation and not on osteoclastogenesis. IL-6 also affects the cartilage metabolism through the SHP/Ras/MAPK signaling pathway; it induces MMP (matrix metalloproteinase) synthesis [57] from chondrocytes, but since it also induces the production of TIMPs (tissue inhibitors of MMPs), IL-6 is thought to play a role in extracellular matrix turnover [58]. Incidentally, in vitro evidence in cultured cells revealed that IL-6 can increase IGFBP-5 mRNA expression in osteoblasts via sIL-6R-mediated mechanisms and it participates also in the development of osteoclasts [59, 60]. The experimental evidence thus suggests that via gp130-mediated signaling pathways, IL-6 and its homologs play important roles in shaping the eventual phenotype of the bone tissues (for details, see a recent review by Sims [61]). In POEMS syndrome, bone lesions have in the majority of cases osteosclerotic character; however, lytic and mixed lesions were also described [11]. Whether bone lesions in POEMS syndrome are related to IL-6 secretion remains speculative. Interestingly, increased levels of IL-6 in subjects with PTHP associated with MG [3, 62] enhanced skeletal sensitivity to the resorbing actions of PTH and led to increased secretion of urine N-telopeptides of type I collagen [63–65].

In thyroid tissue, the action of IL-6 has been examined in conditions other than plasma cell disorder. For example, high IL-6 levels were associated with nonthyroidal illness (NTI) [66–68]. NTI is characterized by low plasma triiodothyronine (T3), low or normal plasma thyroxine (T4), or elevated plasma rT3 in the presence of normal thyrotropin (TSH). Mechanistically, IL-6 suppresses D1- (type I deiodinase-) and D2- (type II deiodinase-) mediated T4-to-T3 conversion and increases D3- (type III deiodinase-) mediated T3 (and T4) inactivation via the MAPK pathway [69] (Figure 2()). Hypothyroidism observed in POEMS syndrome might be related to mechanisms similar to those evidenced in NTI as thyroid function normalized rapidly after hormone replacement treatment and chemotherapy [39]. Interestingly, IL-6 can also mediate destructive processes as observed in cultured thyrocytes; in these cells, increased IL-6 triggered by amiodarone led to destructive thyrocyte lesions [70]. Glucocorticoids appeared to inhibit such effects [71], as they decreased IL-6 mRNA stability and IL-6 synthesis [72]. These observations provide the rationale for the well-known glucocorticoid treatment in patients with amiodarone-induced destructive thyrotoxicosis.

Further, as detailed in Table 4, gonadal axis can be disrupted on multiple levels via the action of proinflammatory mediators. Indeed, various cytokines such as TNF-*α*, IL-6, IL-1*β*, IL-2, and CRH were shown to alter the steroidogenesis in Leydig cells and granulosa cells, the spermatogenesis, and the integrity of the blood-testis barrier and also to modulate the activity of GnRH neurons in the hypothalamus both in vitro and in vivo (for details, see references in Table 3, [73, 74]). Deregulated gonadal axis is often observed in chronic diseases, and it usually resolves upon normalization of the inflammatory state [75, 76]. We speculate that hypogonadism in the present case is of functional origin since the improvement of his gonadal function has been noted, which is in line with the previous reports [14]. The hypogonadism in POEMS may be related to an altered feedback loop between testosterone (T) levels and gonadotropin secretion or to a direct modulation on the function of GnRH neurons by cytokines [73]. Indeed, functional studies in a GnRH-expressing cell line and in primary hypothalamic neuronal cells show that upon binding to gp130, IL-6 or oncostatin M can activate the MAPK and ERK 1/2 intracellular signaling cascade and lead to increased expression of early regulatory genes including c-fos (marker of neuronal activation), transcription factor Egr-1 (early growth response-1, regulator of cell proliferation and programmed cell death), or GADD45*γ* (regulator of genomic stability and of growth arrest) [77]. Such alterations in GnRH neuron function may disrupt LH/FSH output from the pituitary and consequently impair gonadal steroidogenesis.

In the present case, the etiology of adrenal insufficiency is not clear. The HPA axis could not be retested, because of ongoing treatment with dexamethasone. However, adrenal tissue damage may not account for this presentation for the reasons already mentioned above. Our conclusion is also supported by the finding of a case of spontaneous clinical and biochemical recovery of adrenal insufficiency in a patient with POEMS syndrome [78]. Based on the fact that cytokines are capable of modifying adrenal secretory output in response to stress and inflammation [79–82] and that IL-6R mRNA and IL-6R are expressed in the adrenal medulla [83, 84], we speculate that IL-6 signaling may modulate the reactivity of the HPA axis to stress via altering cortisol-CRH-ACTH feedback loop or adrenal steroidogenesis. This idea is supported by the evidence that (1) under excess and prolonged HPA axis activation, the expression of the inhibitory SOCS protein induced by gp130 cytokine (via JAK/STAT pathway) activation can, in turn, inhibit further corticotroph (via JAK/STAT) signaling and that (2) the application of IL-6 in nanomolar concentration range in adrenal chromaffin cells [85] (comparable to those observed during sepsis [49]) can activate ERK 1/2 and STAT3 pathways and lead to increased expression of target genes such as secreted neuropeptides including galanin, PTHrP, G-protein-coupled receptor (GPR), stanniocalcin-1 (STC1) and hypoxia-inducible factor 1*α* (HIF-1*α*) (Figure 2). Indeed, a high expression of HIF-1*α* has been reported in POEMS syndrome [38]. Possibly, in POEMS patients, the hypoxia-responsive microRNAs such as miR-10b, activated by HIF-1*α*, can act as a negative regulator of the steroidogenic genes like CYP11B1 and CYP11B2 [86], reducing thus the production of stress hormones.

## 6. Concluding Remarks

Symptoms such as hypercalcemia, renal impairment, anemia, bone lytic lesions, lymphadenopathy, and hepatosplenomegaly are generally accepted as part of the diagnosis criteria for plasma cell disorders, but in reality, these typical presentations may be either absent or nonrevealing in particular cases such as MGUS or POEMS, where a rather weak presence of M-protein can easily be overlooked owing to the large abundances of physiological serum proteins. The diagnosis may thus be misled. This was the case for our patient at first. His serum protein electrophoresis (sPEP) was found normal initially. Although we eventually evidenced his abnormal M-protein present in the immunoblot of sPEP after detecting it manually using specific immunoglobulin antisera at various dilutions, at first, we were more focused on treating his very prominent endocrinopathies. This case thus alerted us, the endocrinologists, to be aware of the possible association of important endocrine dysfunction with MG. We thus recommend prescribing sPEP and using nonautomated immunofixation in patients having endocrine abnormalities with unknown etiology or with unusual clinical course. Similarly, in patients presenting monoclonal immunoglobulin without classical signs of progressive MG, endocrine dysfunction may occur. The underlying causes for the dysfunction should thus be probed and possibly treated.

From a broader therapeutic point of view, for patients presenting with endocrine dysfunction associated with plasma cell disorder or chronic proinflammatory state, targeting cytokine signaling may represent an additional therapeutic tool to prevent or reduce further aggravating consequences of the endocrine dysfunction, as not only the productions of proinflammatory cytokines are frequently deregulated in these patients but also the widely distributed cytokine receptors are present in endocrine tissues encompassing the brain, adrenals, thyroid, testis, ovary, and placenta as well as islet *β*-cells. One such example is altered response of the HPA axis to somatic stress that can further be aggravated by associated hypogonadism. Interestingly, in rodents and primates with hypogonadism, the hyperreactivity of the HPA axis due to stress and increased cytokines such as IL-6 was attenuated by sex hormone replacement [87–89]. Similarly, in postmenopausal women, estradiol (E2) replacement alleviated endotoxin-stimulated release of ACTH, cortisol, and cytokines (IL-6, TNF-*α*) [90].

The correction of endocrine dysfunction could thus be beneficial for both the amelioration of cytokine profile and the normalization of hormonal feedback mechanisms. However, further studies are necessary to fully determine the clinical relevance of these experimental data.

## Figures and Tables

**Table 1:** Features of the endocrinopathy and the underlying monoclonal gammopathy.

Clinical entity (underlying PCD) *Study design*	Population (F)/age (yr)	Most prominent features (%) (*n*/total *n* tested)	Mobility/protein subclass (*n*/total *n* tested)	Ref
POEMS syndrome *Retrospective*	99 (37)/51	*Revealing signs* Neuropathy (95%) Endocrinopathy (67%) *Endocrine dysfunction* Erectile dysfunction (44/62) Low testosterone (24/28) Hyperestrogenemia (NR) Gynecomastia (17/62) Hyperprolactinemia (5/25) Diabetes mellitus (3/99) Hypothyroidism (14/99) Hyperparathyroidism (3/4) Adrenal insufficiency (14/35)	M-protein (89/99) IgG*λ* (40/88) IgA*λ* (44/88) *λ* light chain (3/88) IgM*λ* (1/88) Polyclonal Ig (7/99)	[11]

POEMS syndrome *Retrospective*	102 (33)/46	*Revealing signs* Neuropathy (51%) Peripheral edema (12%) Neuropathy + peripheral edema (14%) *Endocrine dysfunction* Erectile dysfunction (39/50) Low testosterone (NR) Hyperestrogenemia (11/19) Gynecomastia (43/63) Hyperprolactinemia (NR) Diabetes mellitus (26/93) Hypothyroidism (5/21) Hyperparathyroidism (NR) Adrenal insufficiency (5/26)	M-protein (76/102) IgG*λ* (38/71) IgA*λ* (29/71) *λ* light chain (NR) IgM*λ* (NR) Polyclonal Ig (11/102)	[9]

POEMS syndrome *Retrospective*	25 (8)/51	*Revealing sign* Neuropathy (84%) *Endocrine dysfunction* Erectile dysfunction (13/13) Low testosterone (9/19) Hyperestrogenemia (4/11) Gynecomastia (10/13) Hyperprolactinemia 4/17 Diabetes mellitus 9/22 Hypothyroidism 10/22 Hyperparathyroidism NR Adrenal insufficiency NR	M-protein (25/25) IgG*λ* (9/25) IgA*λ* (12/25) *λ* light chain (4/25) IgM*λ* (NR) Polyclonal Ig (NR)	[10]

PHPT (MGUS, MM) *Prospective*	PHPT (*n* = 101)/58 (30–92)	↑ calcium ↑ PTH	PHPT (10%) IgG*κ* (*n* = 5) IgM*κ* (*n* = 2) IgA*λ* (*n* = 2)	[3]
	Surgical control (*n* = 127)/60 (40–78)	Normal calcium Normal PTH	Surgical control (2%)^#^ IgG*κ* (*n* = 2)	
	Thyroid control (*n* = 101)/56 (32–89)	Normal calcium Normal PTH	Thyroid control (3%)^##^ IgG*λ* (*n* = 2) IgA*λ* (*n* = 1)	

Hyperprolactinemia (MM) *Retrospective*	MM (*n* = 13) non-MM (*n* = 5)	*Hyperprolactinemia* Circulation: ↑ PRL levels Bone marrow: PRL staining	NR	[91, 92]

Chronic thyroiditis (lymphoplasmacytic lymphoma) *Case report*	61/F	Hypothyroid goiter treated by levothyroxine (N TSH) Dizziness	M-spike IgG*γ* No light chain	[4]
Chronic thyroiditis (plasmacytoma) *Case report*	66/F	Chronic thyroiditis (N TSH)	IgG*β*2/*γ*1	[5]

Chronic thyroiditis (plasmacytoma) *Case report*	65/M	Chronic thyroiditis (↑ TSH)	IgG*γ*/NR	[6]

Chronic thyroiditis (lymphoplasmacytic lymphoma) *Case report*	75/F	Chronic thyroiditis (N TSH)	IgG*β*/NR	[93]

Values are expressed in mean ± SD or median (range); ^#^PHPT group versus surgical group (*P* < .005); ^##^PHPT group versus thyroid group (*P* < .04). BM: bone marrow; IFE: immunofixation electrophoresis; MGUS: monoclonal gammopathy of undetermined significance; MM: multiple myeloma; M-protein: monoclonal protein; N: normal; *n*: number; NR: not reported; PCD: plasma cell disorder; sPEP: serum protein electrophoresis; PHPT: primary hyperparathyroidism.

**Table 2:** Descriptive case: hormonal findings.

	Descriptive case		Reference value
	Before treatment	2 months after treatment start	
*Corticotrope axis*			
Basal ACTH (pg/ml)	120	—	5–60
ACTH stimulation test: peak cortisol (nmol/l)	101	—	>550
*Gonadotrope axis*			
FSH (UI/l)	6.9	5.5	2–12
LH (UI/l)	11	5.6	2–9
Testosterone (*μ*g/l)	2	13	2–11
*Thyreotrope axis*			
TSH (mUI/l)	11	3.2	
FT4 (pmol/l)	9	12	9–19
Antibodies			
(i) Antithyroperoxydase	Negative		
(ii) Antithyroglobulin	Negative		

**Table 3:** Diagnostic criteria of POEMS syndrome according to Dispenzieri^∗^.

*Major criteria* Polyneuropathy Monoclonal plasmaproliferative disorder
*Minor criteria*
Sclerotic bone lesion
Castleman's disease
Organomegaly
Edema (edema, pleural effusion, and ascites)
Endocrinopathy (adrenal, thyroid, pituitary, gonadal, parathyroid, and diabetes^‡^)
Skin changes (hyperpigmentation, hypertrichosis, plethora, hemangioma, and white nails)
Papilledema
*Other signs* Clubbing, weight loss, hyperhidrosis, pulmonary hypertension, thrombotic diatheses, diarrhea, and low vitamin B12

^∗^Two major criteria and at least 1 minor criterion are required for diagnosis [11]. ^‡^Because of the high prevalence of diabetes mellitus and thyroid abnormalities, this diagnosis alone is not sufficient to meet this minor criterion.

**Table 4:** Mechanisms linking cytokines in monoclonal gammopathy to endocrine dysfunction.

Mediator	Clinical context	Biological effects in relation to endocrine function
↑ IL-1*β*	Benign and malignant thyroid disease, NTI [94, 95]	(i) Stimulation of IL-6 expression [49, 94, 95]
		(ii) Induction of NTI in the rat [96]
		(iii) No change in thyroid hormone level in human after blockade of IL-1R [97]
	Adrenal response to stress [79]	(i) Increased release of NPY, NE, and EP from human chromaffin cells via MAPK-dependent mechanism and nitric oxide synthase activation [79]
	Gonadal function	(i) Reduction in LH secretion from the anterior pituitary [98]
	(i) GnRH/LH output	(ii) Inhibition of gonadotropin-stimulated granulosa and Leydig cell steroidogenesis [99, 100]
	(ii) Steroidogenesis	

↑ IL-6 (sIL-6R)	Response of the HPA axis to stress	(i) Stress-induced activation of ERK 1/2 and STAT3 pathways in adrenal chromaffin cells [85]
		(ii) Stimulation of the adrenal cortex during somatic [101, 102] and mental stress [104, 105]
		(iii) Stress-induced activation of the HPA axis via STAT3 [106, 107]
	Pituitary senescence and tumor growth	(i) Increased VEGF production, tumor cell proliferation, and ECM remodeling [108–110]
		(ii) Regulation of normal pituitary cell senescence [35]
	PHPT [64, 65]	(i) Bone remodeling and growth plate closure via STAT and SHP2/MAPK pathways [30, 56]
	Thyroid disease: Thyroiditis [94, 103], NTI [66–68], and thyroid carcinoma [94]	(i) Amiodarone-induced production of IL-6 by thyrocytes [70]
		(ii) Inhibition of thyroid function in the presence of sIL-6R in cultured human thyroid follicles [111]
		(iii) Suppression of D1- and D2-mediated T4-to-T3 conversion and increase in D3-mediated T3 (and T4) inactivation in human cells [69]
	Gonadal function	(i) Suppression of GnRH and/or LH secretion [77]
	(i) GnRH/LH output	(ii) Inhibition of gonadal steroidogenesis [112]; impaired expression of LHR mRNA during maturation of granulosa cells [113]
	(ii) Steroidogenesis	(iii) Inhibition of meiotic DNA synthesis of spermatocytes [114]
	(iii) Spermatogenesis	(iv) Altered Sertoli cell tight junction dynamics via MAPK and ERK cascade [115, 116]
	(iv) BTB permeability	

↑ TNF-*α*	Adrenal medulla response to stress	(i) Increased expression of IL-6 mRNA in cultured bovine chromaffin cells [80, 85]
	Gonadal steroidogenesis	(i) Decreased StAR expression and T synthesis in Leydig cell [117]; decreased aromatase activity in granulosa cells [99]

↑ VEGF (VEGF165)	POEMS syndrome [19, 21, 118, 20]	(i) No correlation between VEGF and thyroid hormone levels or between VEGF and testicular function [14, 39]
	Pituitary tumor growth	(i) IL-6 stimulated an increase in VEGF production [109]

BTB: blood-testis barrier; D1: type I deiodinase; D2: type II deiodinase; ECM: extracellular matrix; EP: epinephrine; IL-1R: IL-1 receptor; MAPK: mitogen-activated protein kinase; MGUS: monoclonal gammopathy of undetermined significance; NE: norepinephrine; NTI: nonthyroidal illness; PGC-1*α*: peroxisome proliferator-activated receptor (PPAR) gamma coactivator 1-alpha; PHPT: primary hyperparathyroidism; sIL-6R: soluble IL-6 receptor; T: testosterone; StAR: steroidogenic acute regulatory protein.
